# Neuroprotection and Functional Recovery Associated with Decreased Microglial Activation Following Selective Activation of mGluR2/3 Receptors in a Rodent Model of Parkinson's Disease

**DOI:** 10.4061/2010/190450

**Published:** 2010-05-23

**Authors:** Hugh Chan, Helen Paur, Anthony C. Vernon, Virginia Zabarsky, Krishna P. Datla, Martin J. Croucher, David T. Dexter

**Affiliations:** ^1^Parkinson's Disease Research Group, Faculty of Medicine, Imperial College London, 4th Floor, Burlington Danes Building, Hammersmith Hospital, Du Cane Road, W12 0NN London, UK; ^2^Centre for the Cellular Basis of Behaviour, Department of Neuroscience, Institute of Psychiatry, Kings College London, 1st Floor, The James Black Centre, Coldharbour Lane, SE5 9NU London, UK

## Abstract

Clinical trials have demonstrated positive proof of efficacy of dual metabotropic glutamate receptor 2/3 (mGluR2/3) agonists in both anxiety and schizophrenia. Importantly, evidence suggests that these drugs may also be neuroprotective against glutamate excitotoxicity, implicated in the pathogenesis of Parkinson's disease (PD). However, whether this neuroprotection also translates into functional recovery is unclear. In the current study, we examined the neuroprotective efficacy of the dual mGluR2/3 agonist, 2*R*,4*R*-4-aminopyrrolidine-2,4-dicarboxylate (2*R*,4*R*-APDC), and whether this is accompanied by behavioral recovery in a rodent 6-hydroxydopamine (6-OHDA) model of PD. We now report that delayed post lesion treatment with 2*R*,4*R*-APDC (10 nmol), results in robust neuroprotection of the nigrostriatal system, which translated into functional recovery as measured by improved forelimb use asymmetry and reduced (+)-amphetamine-induced rotation compared to vehicle treated animals. Interestingly, these beneficial effects were associated with a decrease in microglial markers in the SNc, which may suggest an antiinflammatory action of this drug.

## 1. Introduction

Dopamine replacement therapy provides effective relief of motor symptoms in PD patients, but has no proven effect on the degeneration of the nigrostriatal system [1, 2]. Pathophysiological evidence suggests that neurodegeneration in PD has an excitotoxic component, mediated through hyperactivity of glutamatergic subthalamic nucleus (STN) output pathways as a consequence of striatal dopamine (DA) depletion [3]. Owing to their differential expression and neuromodulatory role in glutamate neurotransmission in the basal ganglia, metabotropic glutamate receptors (mGluR) may represent promising drug targets for PD pharmacotherapy [4, 5]. Theoretically, mGluR are perceived to be advantageous due to the lack of adverse effects that are induced by ionotropic receptor antagonists [6]. This is borne out by recent positive proof of efficacy of Group II mGluR (comprising mGluR2 and mGluR3 subtypes) agonists in clinical trials of both schizophrenia [7] and generalized anxiety disorder [8], in which these drugs were well tolerated with minimal extrapyramidal side effects. Interestingly, group II mGluR are considered promising drug targets for “neuroprotective receptors”, since their activation inhibits glutamate release at key synapses in the basal ganglia, including subthalamonigral synapses that are overactive as a result of striatal DA depletion in PD [9–11]. Further, activation of mGluR2/3 stimulates the production and release of neurotrophic factors from glial cells in vitro [12, 13] and in vivo [14–16]. Results from our laboratory and others reveal a moderate neuroprotective effect of Group II mGluR agonists in rodent models of PD, the magnitude of which was dependent on lesion severity [14, 17–19]. Nevertheless, it remains unclear whether these neuroprotective effects also translate effectively into functional recovery in vivo. Indeed, systemic administration of selective dual mGluR2/3 agonists reverses reserpine-induced akinesia, but not rotational asymmetry following amphetamine challenge in vivo, despite concomitant, albeit weak, neuroprotection of the nigrostriatal system [18]. By contrast, other studies have reported reversal of both haloperidol-induced muscle rigidity and catalepsy in rats following Group II mGluR agonist treatment [9, 20]. On the other hand, intrastriatal injection of the dual mGluR2/3 agonist 2*R*,4*R*-4-aminopyrrolidine-2,4-dicarboxylate (2*R*,4*R*-APDC) had no effect in these behavioural tests [21]. Interestingly, intranigral injection of the nonselective mGluR2/3 agonist DCG-IV alleviated reserpine-induced akinesia in rodents [22]. However, this compound is not selective for mGluR2/3 receptors alone and thus is not appropriate for use with in vivo models [23]. 

Taken together, pharmacotherapy targeting Group II mGluR in the basal ganglia appears to offer the potential to combine both symptomatic improvements with potential neuroprotection of remaining nigral neurons [4, 5]. Nevertheless, there seems to be conflicting data in the extant literature regarding both the degree of neuroprotection and the behavioural effects of dual mGluR2/3 treatment in vivo in animal models of PD, which may in part be related to the differing routes of administration employed in each study. Thus, our goal in the current study was to extend our initial findings with the Group II mGluR agonist 2*R*,4*R*-APDC to address this question in a rodent partial lesion model of PD. In addition, taking into account the published effects of Group II mGluR activation on glial cells, we have examined inflammatory markers, alongside markers of neuronal loss in the nigra to determine whether intranigral treatment with 2*R*,4*R*-APDC also influences inflammatory changes in vivo.

## 2. Experimental Procedures

### 2.1. Materials

2*R*,4*R*-APDC was obtained from Tocris Cookson Bioscience (Bristol, UK). All other reagents and compounds were obtained from Sigma-Aldrich Ltd. (Poole, UK) unless stated otherwise in the text. 

### 2.2. Experimental Animals

Male Sprague-Dawley rats (250 ± 20 g; Harlan UK Ltd., Bicester, UK) were housed in groups of three at 21 ± 1°C on a 12-hour light  :  dark cycle (lights on 07:00 h, lights off 19:00 h). Standard rat chow and drinking water were available *ad libitum* throughout the study. All animal experiments were carried out in accordance with the guidelines published in the Home Office Animals (Scientific Procedures) Act, UK, 1986 under project license number PIL 70/6486, with local ethical approval (Ethical Committee of Imperial College, London).

### 2.3. Stereotaxic Implantation of Guide Cannulae

Rats were anaesthetized with a mixture of isoflurane (5% for induction, 1%–3% for maintenance, flow rate 1 L/min) in medical air/oxygen mixture (70/30%) and placed in a stereotaxic frame (David Kopf instruments, Tujunga, USA), with the incisor bar set at 3.3 mm above the interaural line [24]. Body temperature was monitored using a rectal probe and maintained at 37°C during surgery using a thermostatically controlled heated mat. Stainless steel guide cannulae (26-gauge, Plastics One, Roanoke, VA, USA) were unilaterally implanted 1 mm above the left SNc at the following coordinates; AP: −3.0 mm and ML: +2.5 mm (relative to bregma), DV: −7.6 mm (relative to dura; Paxinos and Watson, 1986) as described previously [19]. Postsurgery, rats were placed in a heated recovery chamber to recover from the anaesthetic. Postoperative care included individual caging, analgesia (buprenorphine, 0.3 mg/kg s.c during the first 48 hours), fluid replacement (4 mL 0.18% glucosaline solution i.p), and mashed high-nutrient food pellets during the first week after surgery. Animals were checked daily for signs of gross neurological or behavioural abnormalities and weighed to monitor recovery. No experimental interventions were made until animals had completed a minimum 10-day recovery period.

### 2.4. Experimental Design

Following recovery from surgery, cannulated animals were randomly assigned to drug (*n* = 8) or vehicle treatment groups (*n* = 10). To investigate the effects of delayed 2*R*,4*R*-APDC-treatment on neuroprotection, 6-OHDA lesioned animals received intranigral injections of 10 nmol 2*R*,4*R*-APDC or drug vehicle, once daily, for seven consecutive days as per our previously published protocol [19] with the exception that drug treatment was delayed until 48 hours post lesion. To evaluate whether 2*R*,4*R*-APDC improved the behavior of 6-OHDA lesioned animals, both treated and control animals were assessed for their performance in the spontaneous forelimb test and the degree of contralateral rotation following amphetamine injection. Performance in the forelimb asymmetry test was assessed prior to lesioning and drug treatment to establish a baseline and control for the effects of cannulae implantation on behavior. Animals were retested for performance in this test upon completion of drug treatment (7 days), equivalent to 9 days post lesioning. Animals were assessed for amphetamine-induced rotational behavior 24 hours later. In both treatment groups, neuroprotection was quantified by postmortem immunohistochemical analysis of tyrosine hydroxylase (TH)-positive cells in the substantia nigra pars compacta (SNpc) and biochemical measurement of strital monoamine concentrations by HPLC. In addition neuronal cell counts were performed in the SNc to control for changes in TH expression in atrophic neurons. Lastly, the effects of 6-OHDA lesioning and drug or vehicle treatment on inflammatory markers were assessed using postmortem immunohistochemistry for astroglial and microglial cell markers, respectively. Details of 6-OHDA lesioning, drug preparation and administration, behavioral testing, and postmortem immunohistochemical, and biochemical analyses are provided in the sections below.

### 2.5. Induction of 6-OHDA Nigrostriatal Lesions

Unilateral 6-OHDA nigrostriatal lesions were created as previously described elsewhere [25]. Briefly, 12 *μ*g 6-hydroxydopmine hydrobromide (6-OHDA, free base, Sigma-Aldrich Ltd. Poole, UK) in 4 *μ*L 0.1% ascorbic acid/saline solution was infused into the left SNc using a 26-gauge stainless steel injection cannulae, extending 1 mm below the tip of the indwelling guide cannula, attached with flexible tubing (Portex, Hythe, UK) to a 10 *μ*L 700 series Hamilton syringe mounted on a motorised Harvard micropump (Harvard Apparatus, Edenbridge, UK). Infusions were made at a rate of 1 *μ*L/min followed by a 5-minute equilibration time, during which the needle remained in place and was then slowly retracted. 

### 2.6. Drug Preparation and Intranigral Administration

Stock solutions of 2*R*,4*R*-APDC were prepared in phosphate buffered saline (PBS; mM: NaCl 137; KCl 2.7; KH_2_PO_4_ 1.8; Na_2_HPO_4_ 10) and adjusted to pH 7.4. Drug concentrations were selected based on published in vitro EC_50_ and *K*_*i*_ values for each compound at mGluR2/3 [23, 26] and on our previous experience with this compound [19] to avoid confounding effects due to activation of other mGluR subtypes. 2*R*,4*R*-APDC was chosen for use as it retains selectivity for mGluR2/3 even at relatively high concentrations [26]. Drugs were aliquoted and stored at −80°C according to the manufacturer's instructions. Fresh drug aliquots were used on each day of drug administration to avoid repeated freeze-thaw cycles. In all treatment groups, unilateral intranigral drug injections were performed exactly as described for 6-OHDA lesions, in a final injection volume of 4 *μ*L. Importantly, in our previous study with this compound [19], drug treatment was initiated one hour prior to 6-OHDA lesioning, which whilst providing robust proof-of-concept, is not ideal. To address this limitation, in the current study drug treatment initiation was delayed until 48 hours post lesion, at which point nigrostriatal degeneration should be ongoing in vivo. In all groups, drug or vehicle treatment was initiated 48 hours post 6-OHDA lesioning. Intranigral injections were performed once daily, at the same time, over 7 consecutive days. Following all intranigral injections, animals were observed qualitatively over a period of 20 minutes for any signs of gross behavioral abnormalities in response to the drug injection. 

### 2.7. Assessment of Spontaneous Forelimb Use and Rotational Asymmetry Following (+)-Amphetamine Challenge

Performance in the spontaneous exploratory forelimb use test was assessed as previously described by others [27, 28]. In brief, animals were placed in a clear plastic cylinder (height: 30 cm, width: 20 cm) and forelimb use asymmetry was assessed during rearing behaviour [27]. Forelimb contact with the cylinder wall was scored as left, right, or both and the percentage forelimb asymmetry was determined using the following formula: ((contralateral limb placements − ipsilateral limb placements)/total placements) × 100) [27]. As described above, forelimb asymmetry was assessed 10 days after cannulation and 1 day prior to 6-OHDA lesioning. Animals were then retested prior to sacrifice, following 7 days of drug treatment, equivalent to 9 days post lesion, by an investigator blinded to the treatment group. 

Rotational asymmetry was assessed 24 hours after completion of drug treatment, equivalent to 10 days post lesion. Animals were administered amphetamine (5 mg/kg i.p.) to induce rotational behavior as described previously [29]. Animals were placed in a clear circular test arena (25 × 15 cm) and following a short period of acclimatization (30 minutes), injected with the dopaminergic agonist. The complete number of contralateral and ipsilateral turns was then recorded for 60 minutes postinjection.

### 2.8. Tissue Preparation

Upon completion of behavioral testing, 9 days after the initial drug injection and 11 days post lesion, animals were sacrificed by decapitation and the brains quickly dissected out onto a chilled platform and cut at the level of the infundibular stem (−4.16 mm from bregma) to produce a fore- and hindbrain block containing the corpus striatum and SNc, respectively. Hindbrain blocks were fixed in 4% paraformaldehyde for 5 days, cryoprotected in 30% sucrose for 24–48 hours and stored desiccated at −80°C until cryostat sectioning. Coronal sections (20 *μ*m-thick) were cut on a cryostat, (Bright Instruments, Cambridge, UK), throughout the rostral to caudal extent of the SNc (−4.80 to 6.30 mm from bregma; Paxinos and Watson, 1986). Free-floating sections were collected in series and stored in PBS (pH 7.4) containing 0.05% sodium azide as a preservative until immunostaining. From the forebrain blocks, the left and right striata were quickly dissected out, snap-frozen, and stored at −80°C until biochemical analysis of monoamine content. 

### 2.9. Immunohistochemistry

Immunohistochemistry was performed using a standard immunoperoxidase method as previously described [25]. Briefly, free-floating sections were washed in PBS (pH 7.4, 3 × 5 minutes) and endogenous peroxidase activity quenched by incubation in 1% hydrogen peroxide solution (30 minutes) followed by cell permeabilisation in PBS containing 0.1% triton X-100 (15 minutes). Nonspecific biding was blocked by incubating sections in 3% normal goat serum (NGS, MP Biomedicals, Eschwage, Germany) diluted in PBS for 1 hour. Sections were incubated with primary antibody diluted in PBS containing 0.1% triton and 3% NGS for 18 hours at room temperature. Dopaminergic neurons were identified using rabbit antirat tyrosine hydroxylase (TH, AB151, Chemicon Europe, Watford, UK, 1  :  3000) with adjacent sections probed with mouse antirat neuron specific nuclear protein (NeuN, MAB377, Chemicon Europe, 1  :  2000;) to control for changes in TH protein expression. Astrocytes were identified using rabbit antirat glial fibrillary acidic protein (GFAP, AB5804, Chemicon Europe, 1  :  400), and two antibodies, mouse antirat cd11b (clone OX-42; CBL1512, Chemicon, Europe, 1  :  400) and mouse antirat major histocompatibility complex (MHC) class II (clone OX6; ABD Serotec, Kidlington, UK) were used to identify microglia and activated microglia, respectively, since it has been reported that activated microglia express high levels of MHC class II [30]. After treatment with primary antibodies, sections were incubated with biotinylated secondary antibody (goat *α*-rabbit or *α*-mouse IgG, Vector Labs, Peterborough, UK) diluted 1  :  200 in PBS for 2 hours. Sections were incubated for a further 1 hour in horseradish peroxidase conjugate (Vectastain Elite ABC Kit, Vector Labs, Peterborough, UK), and antibody binding was visualised using a 3′3-diaminobenzidine (DAB) peroxidase staining kit with nickel enhancement (DAB peroxidase kit SK-4100, Vector Labs, Peterborough, UK) according to the manufacturer's instructions, followed by washing in distilled water. Each step was preceded by washing in PBS (pH 7.4, 3 × 5 minutes). Free-floating immunostained sections were mounted onto poly-L-lysine-coated slides (VWR, Lutterworth, UK) and allowed to adhere by air-drying. Mounted sections were rinsed with tap water before dehydration through a series of graded alcohol and xylene solutions, prior to application of coverslips using DPX mounting medium. Negative controls were performed in which the primary antibody was omitted and in which no immunostaining was observed, thereby confirming the specificity of the primary antibodies. 

### 2.10. Cell Counting

The total number of TH-positive cells were counted manually rostro-caudally through the SNc in contiguous sections using a Nikon Eclipse E800 microscope (Nikon, Tokyo, Japan) connected to a JVC analogue camera and Image Pro Plus software (Media Cybernetics, Finchampstead, UK). The SNc was outlined using a manually traced region of interest (ROI) at low magnification (×4). The number of TH positive cells was counted in the contralateral and ipsilateral hemispheres at the level of third cranial nerve, within a 100 *μ*m × 100 *μ*m counting area at high magnification (×40) only within this defined ROI. Importantly, the level of the third cranial nerve provides a robust anatomical landmark where the SNc can be reliably delineated from the VTA, as previously described [31, 32]. Lesion size was then calculated as a percentage of the unlesioned contralateral hemisphere. Although not a stereological procedure, previous studies have shown that the 3rd nerve rootlets provide a reliable anatomical landmark at which the extent of cell loss is reflective of cell loss throughout the entire substantia nigra [32]. Further, manual cell counts assessed at the level of the third cranial nerve have been demonstrated to give equivalent results, not significantly different to that obtained from unbiased stereological estimates at the same level using an optical fractionator probe design [31]. These data strongly suggest that manual cell counting at the level of the third nerve is a viable method of determining cell loss and neuroprotection in this model [31]. 

To control for changes in TH expression in atrophic neurons, NeuN+ cell bodies in the SNc were counted in exactly the same manner as described above with reference to adjacent TH-immunostained sections from the same animal. Two operators blinded to the treatment group under analysis performed all quantitative cell counting (H.C. and H.P.). At the same time as quantitative analysis was performed, representative photomicrographs were captured at ×40 magnification using the same microscope and camera set-up and Image Pro Plus v.5.0 image analysis software (Media Cybernetics, Finchampstead, UK). 

### 2.11. Measurement of Striatal Monoamines Using HPLC-ECD

Striatal monoamine content was analysed using HPLC-ECD, as previously described [19]. Briefly, the left and right striata were thawed, weighed and homogenised in 500 *μ*l of ice-cold homogenisation buffer (50 mM trichloroacetic acid, 0.5 mM EDTA) containing 0.5 pmol/mL 3,4-dihydroxybenzylamine hydrobromide (DHBA) as an internal standard. Striata were homogenised for 20 seconds by sonication (Soniprep, Sanyo, Loughborough, UK) and placed on ice for 10 minutes to allow complete extraction of monoamines, followed by centrifugation at 13000 g at 4°C for 10 minutes (Heraeus Centrifuges, Newport Pagnell, UK). Sample supernatant was filtered (0.2 *μ*m PTFE filter, Whatman, Maidstone, UK) into HPLC vials (Chromacol, UK) and loaded onto an autosampler (Gina 50, Dionex, Camberley, UK) maintained at 5°C. From each sample, 20 *μ*L were injected and analysed for dopamine (DA), dihydroxyphenylacetic acid (DOPAC), and homovanillic acid (HVA) content using a phosphate buffer mobile phase (0.1 mM KH_2_PO_4_, 0.1 mM EDTA, and 1 mM octyl sodium sulphonate, 10% methanol V/V, adjusted to pH 2.5 with orthophosphoric acid) at a flow rate of 0.9 mL/min on an Altex Ultrasphere 3 *μ*m ODS column (4.6 mm × 7.5 cm, Beckman-Coulter, High Wycombe, UK). Samples were quantified by an electrochemical analytical cell (model 5011, ESA Analytical, Aylesbury, UK) attached to a Coulochem II electrochemical detector (ESA Analytical, Aylesbury, UK) with electrode one set at −0.20 mV and electrode two set at +0.34 mV with respect to the palladium reference electrode online with a dedicated PC-based data analysis programme (Chromeleon, Dionex, Camberley, UK). A set of standards for each monoamine and their metabolite were analysed after every fifth brain sample. Striata from both control and mGluR agonist-treated groups were analysed on the same day. 

### 2.12. Statistical Analysis of Data

Data from vehicle and drug-treated groups were compared using two-tailed Student's *t*-test in Prism v5.0 software (GraphPad software, San Diego, CA, USA). All data are presented as mean ± standard error of the mean (s.e.m) and differences were considered statistically significant at *P* < .05.

## 3. Results

### 3.1. Delayed Intranigral Injection of 2R,4R-APDC Provides Robust Neuroprotection against 6-OHDA Toxicity

**Figure 1:**
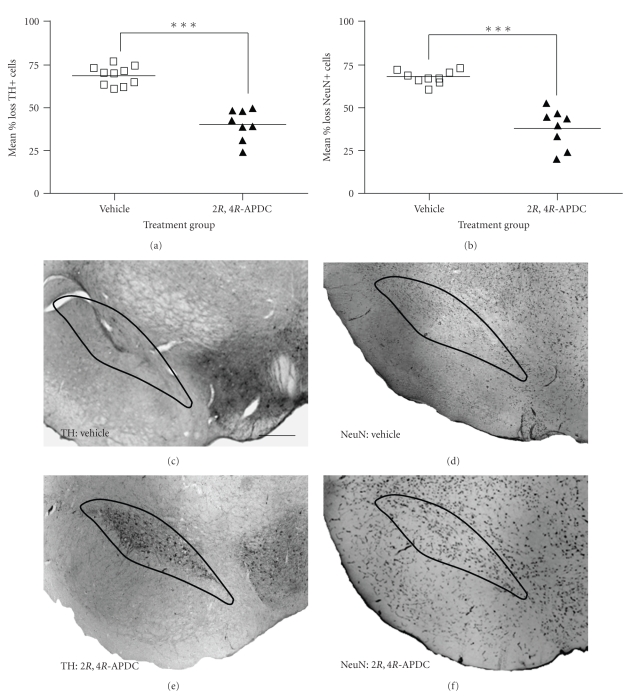
Delayed (48 hours post lesion) subchronic intranigral administration of 2*R*,4*R*-APDC (10 nmol in 4 *μ*L) attenuates 6-OHDA toxicity in vivo. Scatter plots showing (a) the mean percentage loss ± s.e.m of nigral TH+ cells and (b) NeuN+ cells in each treatment group (Vehicle *n* = 10, 2*R*,4*R*-APDC, *n* = 8). Note that 6-OHDA induces an equivalent reduction of both TH and NeuN+ cells in the SNc, which is significantly attenuated following delayed administration of 2*R*,4*R*-APDC (10 nmol). ***P < .001, vehicle versus 2*R*,4*R*-APDC (2-tailed students *t*-test). (c)–(f) Representative photomicrographs (×40) of DAB/peroxidase staining for TH and NeuN+ cells in the SNc from each treatment group, also showing the ROI encompassing the SNc used for cell counting, scale bar 200 *μ*m. (c) TH, 6-OHDA + vehicle, (d) NeuN,6-OHDA + vehicle, (e) TH, 6-OHDA + 2*R*,4*R*-APDC, (f) NeuN, 6-OHDA + 2*R*,4*R*-APDC.

The mean number of TH+ cells in the unlesioned hemisphere of drug or vehicle-treated animals was comparable and not significantly different. Intranigral injection of 6-OHDA (12 *μ*g) resulted in a ~70% reduction (68.75 ± 1.71 [95% CI: 64.89%–74.62%] of TH+ cells in the ipsilateral SNc of vehicle-injected-animals (*n* = 8) relative to the contralateral hemisphere; Figure 1(a)). By contrast, in animals receiving intranigral injections of 2*R*,4*R*-APDC (10 nmol) for 7 days, starting 48 hours post lesion, there was a significantly smaller reduction in TH+ cells in the lesioned SNc compared to vehicle treated animals (40.26 ± 3.18% [95% CI: 32.74%–47.78%] versus 68.75 ± 1.71% [95% CI: 64.89%–74.62%]; *P* < .001; Figure 1(a)). To control for possible alterations in TH expression within atrophic neurons influencing the observed effects on TH+ cell number in the SNc, additional cell counts were performed in the same animals from each treatment group on tissue sections stained with the specific neuronal marker, NeuN. Critically, the loss of TH+ cells in the SNc was mirrored by an equivalent reduction in NeuN+ cells in the lesioned SNc in vehicle-treated animals (67.43 ± 1.15% [95% CI: 64.74%–69.95%]). By contrast, a significantly smaller reduction in NeuN+ cells was observed in 2*R*,4*R*-APDC-treated animals compared to vehicle-treated animals (37.89 ± 4.03% [95% CI: 28.36%–47.42%] versus (67.43 ± 1.15% [95% CI: 64.74%–69.95%]; *P* < .001; Figure 1(b)), consistent with the observed preservation of TH+ cells in the lesioned SNc. These data are illustrated in representative photomicrographs of nigral TH+ and NeuN+ cells from both groups in Figures 1(c)–1(f). 

No significant changes in monoamine content or turnover were observed in sham-lesioned animals (Table 1). Biochemical analysis of monoamine levels in the corpus striatum in each treatment group demonstrated that the neuroprotective effects of delayed 2*R*,4*R*-APDC treatment observed at the cellular level were associated with moderate, but statistically significant preservation of DA, DOPAC, and HVA concentrations in the ipsilateral corpus striatum compared to vehicle-injected controls (*P* < .05; Table 1). Importantly, no significant differences were observed for any monoamine concentration in the contralateral hemisphere between vehicle and drug-treated animals (*P* > .05; Table 1). A significant increase in striatal dopamine turnover ratio was observed in the lesioned striata of vehicle treated animals (*P* < .05; Table 2), indicative of increased DA metabolism as an intrinsic compensatory response to the 6-OHDA lesion. Interestingly, in 2*R*,4*R*-APDC-injected animals, striatal DA turnover remained elevated in the lesioned hemisphere relative to the unlesioned side (*P* < .05; Table 2), but importantly, this was significantly lower than that observed in the lesioned striatum of vehicle-treated animals (*P* < .05; Table 2).

### 3.2. Functional Recovery of Motor Behavior In Vivo Following Delayed Intranigral Injection of 2R,4R-APDC

**Figure 2:**
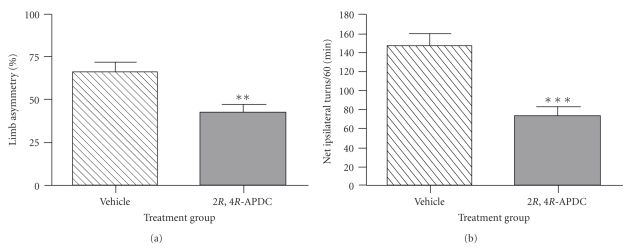
Neuroprotection against 6-OHDA toxicity following delayed subchronic intranigral administration of 2*R*,4*R*-APDC (10 nmol in 4 *μ*L) translates into functional recovery of motor deficits. (a) Bar graph of mean percentage forelimb use asymmetry ± s.e.m in vehicle and 2*R*,4*R*-APDC (10 nmol)-treated animals, **P < 0.01, vehicle versus 2*R*,4*R*-APDC. (b) Bar graph of amphetamine-induced rotational behavior, expressed as net ipsilateral turns over 60 minutes ± s.e.m in vehicle and 2*R*,4*R*-APDC-treated animals, ***P < .001, vehicle versus 2*R*,4*R*-APDC.

**Figure 3:**
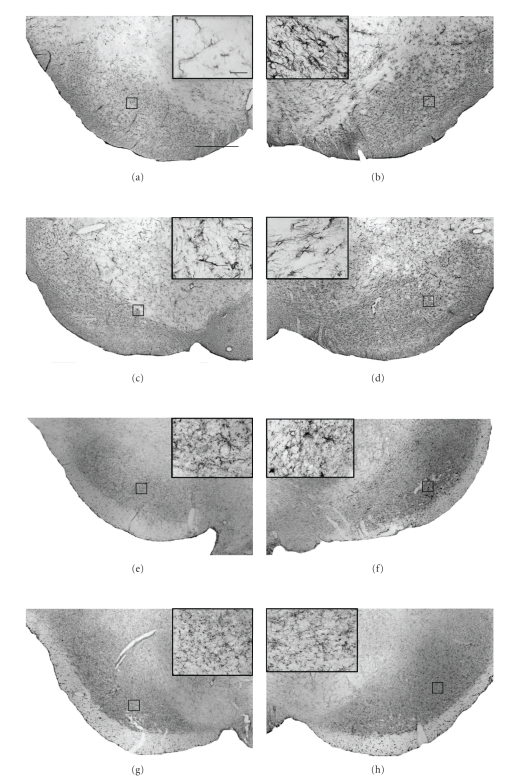
Neuroprotection against 6-OHDA toxicity following delayed subchronic intranigral administration of 2*R*,4*R*-APDC is associated with qualitative reductions in inflammatory markers. Representative photomicrographs (×40) of DAB/peroxidase staining for GFAP (astrocytosis) and OX-42 (microgliosis) in the SNc from each treatment group (scale bar 200 *μ*m.). Panels (a)–(g) show the intensity and distribution of GFAP+ and OX-42+ cells in the contralateral control SN of vehicle (a), (c) and (e), (g) 2*R*,4*R*-APDC (10 nmol)-injected animals. Panels (b)–(h) show the intensity and distribution of GFAP+ and OX-42+ cells in the Lesioned SNc of vehicle (b), (d) and (e), (g) 2*R*,4*R*-APDC (10 nmol)-injected animals. (Insets (a)–(h)), ×60, enlargements of outlined areas (scale bar 10 *μ*m)). Note the apparent reduction in intensity of GFAP+ and OX-42+ staining in 2*R*,4*R*-APDC-injected animals compared to vehicle-treated animals. Further, 2*R*,4*R*-APDC treatment appeared to reduce the number of cells with morphology characteristic for reactive astrocytes or activated microglia in the SNc in comparison to vehicle-treated controls.

To functionally assess the neuroprotective effect of delayed intranigral 2*R*,4*R*-APDC injection (10 nmol), spontaneous motor performance was assessed by observing forelimb use during exploratory rearing behaviour in the cylinder test. No significant forelimb use asymmetry was detected between treatment groups prior to 6-OHDA lesioning (*data not shown*). Both 2*R*,4*R*-APDC and vehicle-treated 6-OHDA lesioned animals displayed a significant shift towards use of the ipsilateral forelimb relative to the contralateral forelimb. However, this ipsilateral forelimb use bias was moderately, but significantly reduced in 2*R*,4*R*-APDC-injected animals compared to vehicle-treated animals (42.6 ± 4.1% [95% CI: 32.79%–52.41%] versus 66.4 ± 5.9% [95% CI: 52.78–80.07]; *P* < .05; Figure 2(a)). In addition, the ipsilateral rotational responses to (+)-amphetamine were also investigated just prior to sacrifice. Injection of 5 mg/kg (+)-amphetamine (i.p.) evoked a significant increase in net ipsilateral turns over the 60-minute recording session in vehicle-treated 6-OHDA lesioned animals (Figure 3(b)), which was markedly attenuated in animals receiving delayed intranigral 2*R*,4*R*-APDC (*P* < .001; Figure 2(b)).

### 3.3. Effect of Delayed Treatment with 2R,4R-APDC on Expression of Astroglial and Microglial Markers in the Substantia Nigra Pars Compacta

**Figure 4:**
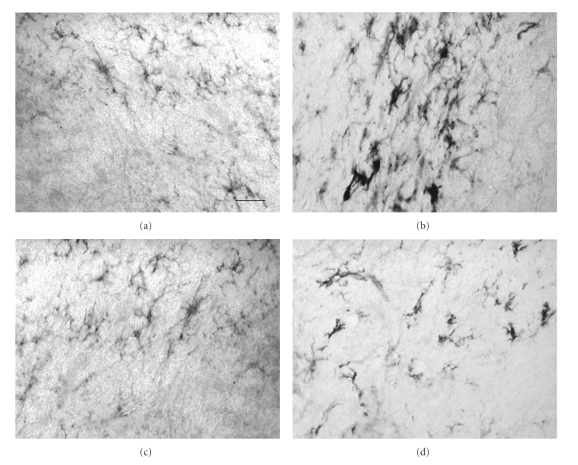
Neuroprotection against 6-OHDA toxicity following delayed subchronic intranigral administration of 2*R*,4*R*-APDC is associated with reduced numbers of activated microglia in the SNc. Representative photomicrographs (×40) of DAB/peroxidase staining for OX-6, a marker of activated microglia in the SNc from each treatment group (scale bar 200 *μ*m.). (a) 6-OHDA + vehicle, unlesioned SNc, (b) 6-OHDA + vehicle, lesioned SNc, (c) 6-OHDA + 2*R*,4*R*-APDC (10 nmol) unlesioned SNc, (d) 6-OHDA + 2*R*,4*R*-APDC (10 nmol) lesioned SNc. Note the increase in intensity of OX-6 staining and the increase in OX-6+ cells displaying morphology of activated microglia in the lesioned SNc of vehicle-treated animal, which appears markedly reduced in animals treated with 2*R*,4*R*-APDC.

In vehicle-treated animals, dopaminergic neuronal death induced by 6-OHDA lesioning was accompanied by significant neuroinflammation. Indeed, compared to the intact unlesioned hemisphere (Figure 3(a)) a marked increase in the intensity of both GFAP and OX-42-immunostaining was observed in the lesioned SNc relative to the unlesioned hemisphere (Figure 3(b)). Both GFAP- and OX-42-positive (GFAP+/OX42+) cells in the unlesioned hemisphere displayed morphology typical of resting or quiescent cells (insets, Figures 3(a) and 3(e)). By contrast, in the lesioned hemisphere, a marked increased in GFAP+ and OX42+ cells displaying morphology typical of activated cells was observed, with astrocytes displaying thickened processes and a switch from ramified to ameboid morphology in microglia (insets, Figures 3(b) and 3(f)). Qualitative analysis suggests that delayed treatment with 2*R*,4*R*-APDC reduced the intensity of GFAP staining in the lesioned SNc, (Figures 3(c) and 3(d)) compared to vehicle-treated 6-OHDA lesioned animals (Figures 3(a) and 3(b)). In addition, fewer GFAP+ cells displayed activated morphology in the lesioned SNc of 2*R*,4*R*-APDC-treated animals compared to vehicle treated 6-OHDA lesioned animals (Figures 3(a)–3(d)). In addition, a clear decrease in the intensity of OX42+ staining in the lesioned SNc could be observed in 2*R*,4*R*-APDC injected animals (Figures 3(g) and 3(h)), compared to vehicle-treated 6-OHDA lesioned animals (Figures 3(e) and 3(f)). Notably, qualitative analysis suggested a marked decrease in OX42+ cells displaying reactive morphology compared to vehicle-treated animals, although this was not completely abrogated (Figures 3(e) and 3(h)). Consistent with these observations, a clear increase in OX6+ cells displaying reactive morphology was also observed in the lesioned SNc of vehicle-treated 6-OHDA lesioned animals compared to the unlesioned hemisphere (Figures 4(a) and 4(b)), confirming the presence of activated microglia. Importantly, this was markedly, but not completely reduced in animals injected with 2*R*,4*R*-APDC (Figures 4(c) and 4(d)).

## 4. Discussion

In the current study we have extended our initial preliminary observations [19] in vivo using the dual mGluR2/3 agonist 2*R*,4*R*-APDC in a rodent partial lesion 6-OHDA model of PD. Indeed, we now report that delayed (48 hour post lesion) subchronic intranigral administration of 2*R*,4*R*-APDC (10 nmol) protects the rodent nigrostriatal system against 6-OHDA toxicity at the cellular (preservation of nigral TH+ cell bodies) and biochemical level (preservation of striatal monoamine concentrations). This is effectively translated into functional recovery as demonstrated by improved forelimb asymmetry scores and correction of amphetamine-induced rotational asymmetry. In addition, these effects were associated with a qualitative decrease in microglial activation in the SNc. Importantly, in animals receiving 2*R*,4*R*-APDC post lesion, NeuN+ and TH+ cell loss was attenuated in parallel in the ipsilateral SNc, strongly supporting a neuroprotective effect, rather than alterations in TH expression in atrophic neurones. However, although intranigral treatment with 2*R*,4*R*-APDC lowered the intrinsic compensatory increases in striatal DA metabolism compared to vehicle-treated animals, this was not completely reversed. Thus, we cannot exclude the possibility that the neuroprotective effects of 2*R*,4*R*-APDC on striatal DA and its metabolites is not partly due to intrinsic compensatory mechanisms. Furthermore, it will be important in future studies to determine whether these data are replicated upon systemic administration and in a more progressive model of PD, such as the intrastriatal 6-OHDA model [33]. Notably, 2*R*,4*R*-APDC did not completely prevent 6-OHDA toxicity in vivo, consistent with previous in vivo studies [14, 17–19]. 

Preliminary data from our laboratory have shown the neuroprotective effects of 2*R*,4*R*-APDC are abrogated by coadministration with EGLU, a highly selective group II mGluR antagonist [34]. These observations strongly suggesting the beneficial effects of 2*R*,4*R*-APDC effects are mediated through selective activation of mGluR2/3 in vivo (Vernon et al., *unpublished observations*). Interestingly, the relatively weak neuroprotective effects observed in previous studies [14, 17–19] may be explained by evidence suggesting that mGlu2 and 3 receptors have divergent functions in vivo [15]. Indeed, in an elegant knock-out mice study, Corti and colleagues (2007) demonstrated that the systemic administration of the dual mGluR2/3 agonist LY379268 protected striatal neurons against NMDA toxicity in wild-type and mGluR2^−/−^ mice but not in mGluR3^−/−^ mice [15]. Moreover, LY379268 was neuroprotective against nigrostriatal degeneration induced by low doses of 1-methyl-4-phenyl-1,2,3,6-tetrahydropyridine (MPTP) in mGluR2^−/−^ mice, strongly suggesting that these in vivo neuroprotective effects are dependent on the activation of mGluR3 [15]. Since LY379268 was only neuroprotective in mGlu2^−/−^ mice, these data suggest that activation of mGluR2 in wild-type mice may counterbalance the protective activity of the drug [15]. This hypothesis is supported by detailed in vitro experiments in the same study, which provided evidence that activation of mGluR2 may enhance NMDA toxicity in mixed cortical cultures [15]. Thus, in vitro and in vivo evidence converges to suggest that mGluR3 mediates the neuroprotective activity of mGluR2/3 receptor agonists in vivo and that a combined activation of mGluR2 and mGluR3 receptors may therefore limit the extent of neuroprotection observed [15]. Conversely, it is interesting to note that mGluR2 activation alone is required for novel antipsychotic actions of dual mGluR2/3 agonists in vivo [35, 36]. However, it is clear that dual mGluR2/3 agonists such as LY367385 and indeed, 2*R*,4*R*-APDC might be predicted to have less neuroprotective efficacy than mGluR3 agonists alone [15]. 

This also has implications for the potential antiparkinsonian effects of these compounds in preclinical models of PD. In the current study, we provide preliminary evidence that neuroprotection following delayed, subchronic intranigral administration of 2*R*,4*R*-APDC translated into functional recovery, confirming a neuroprotective effect at the behavioural level. These data are somewhat consistent with previous data in which *systemic *administration of the dual mGluR2/3 agonist LY379268 weakly ameliorated reserpine-induced akinesia, but did not reverse amphetamine rotational asymmetry in 6-OHDA lesioned rats, despite modest neuroprotective effects [18]. Interestingly, *intrastriatal* injection of 2*R*,4*R*-APDC did not reverse haloperidol-induced catalepsy or muscle rigidity in rats [21]. These divergent results may be explained by data demonstrating that dual mGluR2/3 agonists, (including LY379268 and 2*R*,4*R*-APDC) inhibit not only glutamate, but also DA release in the striatum in vivo [37]. Interestingly, LY379268 also displays partial agonist activity at DA D_2_ receptors in vitro and in vivo [38, 39]. Thus, following systemic or intrastriatal administration, dual mGluR2/3 agonists may bind to presynaptic DA D_2_ receptors thereby decreasing DA release in the striatum and inhibiting locomtion [38, 39]. Whether 2*R*,4*R*-APDC has partial agonist activity at DA D_2_ receptors is unknown. However, recent data suggest that Group II mGluR, in particular mGluR2, are closely associated with DA D_2_ receptors and may regulate their number and activity in the striatum [40, 41]. Indeed, the number of D_2_ receptors in the striatum is markedly elevated in mGluR2 and mGluR3 knock-out mice [40, 41]. Moreover, in vitro striatal homogenates from mGluR2 knock-out mice displayed significantly greater supersensitivity responses to the D_2_ receptor agonist (+)PHNO when compared to those from mGluR3 knock-outs, suggesting that mGluR2 and DA D_2_ receptors are functionally linked in vivo [40]. 

Taken together, these data suggest that translation of neuroprotection into functional recovery following dual mGluR2/3 agonist treatment is critically dependent on the route of drug administration. Indeed, our own data demonstrate that this may be observed following activation of nigral mGluR2/3 in isolation. By contrast, following systemic administration, confounding actions in other brain regions particularly, but not exclusively, in the striatum might occlude this effect [18]. Notably again, this appears predominantly due to the activation of mGluR2 [38]. Thus, these data reinforce the prediction that mGluR3 selective compounds may be more efficacious as both neuroprotective and antiparkinsonian agents, although this remains to be tested in vivo. 

In the current study we also observed that the neuroprotective effects of 2*R*,4*R*-APDC were associated with a qualitative decrease in microglial markers in the SNc. Interestingly, in vitro, activation of mGluR3 expressed in microglia inhibits microglial activation and secretion of neurotoxic cytokines [42, 43]. Conversely, activation of microglial mGluR2 enhances microglial toxicity in vitro [42, 43]. Again, these data are consistent with divergent roles of mGluR2 and 3 in vivo [15] and may certainly also contribute to the modest neuroprotective effects of 2*R*,4*R*-APDC in vivo by counterbalancing beneficial effects of mGluR3 activation. Nevertheless, based on this observation it is tempting to speculate that the neuroprotective effects of 2*R*,4*R*-APDC and other dual mGluR2/3 agonists in vivo may involve antiinflammatory mechanisms, including prevention of toxic microglial activation, alongside documented effects such as promoting secretion of trophic-factors from astrocytes [12–15]. However, these data should be interpreted cautiously, as decreases in microglial markers may simply reflect a secondary effects due to neuroprotection by 2*R*,4*R*-APDC through alternative mechanisms. Nevertheless, these initial observations suggest that further studies on the effects of mGluR2/3 agonists on microglial response in vivo may be warranted.

## 5. Conclusions

In conclusion, this study extends our preliminary findings [19] and provides evidence that subchronic intranigral injection of the dual mGluR2/3 agonist 2*R*,4*R*-APDC robustly attenuates dopaminergic neurodegeneration in vivo, even when treatment initiation is delayed. Furthermore, this translates into modest functional recovery, at least following *intranigral *administration in vivo. Moreover, the neuroprotective effects of 2*R*,4*R*-APDC may involve modification of the inflammatory response as evidenced by a qualitative reduction on the expression of microglial markers in the SNc. However, we fully acknowledge that the significance of these data should be interpreted with the caveat that we have utilised a dual mGluR2/3 agonists and thus any beneficial effects are likely to be influenced by confounding effects due to activation of mGluR2 in vivo [15]. Nevertheless, these data provide further evidence for the rationale of targeting Group II mGluR in PD, although it will be vitally important in future studies to repeat these experiments using mGluR3-selective compounds as these become available. Overall, this also has implications for the clinical use of dual mGluR2/3 agonists for the treatment of neurodegenerative diseases. Indeed, on the basis of currently available data from pre-clinical studies, orthosteric nonselective mGluR2/3 agonists may be of limited clinical use as neuroprotective/antiparkinsonian agents due to confounding effects at mGluR2 in vivo [15]. The development of a novel class of compounds, known as positive allosteric modulators (PAMs), which do not activate the receptor directly, but bind to a site distinct from the glutamate-binding site, with the effect of increasing the response of the receptor to endogenous glutamatergic tone, may offer a solution to this problem, since such binding sites are often less constrained and more amenable to discovery of novel, subtype-selective compounds, compared to targeting the glutamate binding site [44]. Indeed, a number of PAMs selective for mGluR2 have been developed, which may offer an exciting alternative approach to dual mGluR2/3 agonists at least for their antipsychotic or anxiolytic indications [44–47]. Notably however, this has not been matched by a similar development of mGluR3 selective PAMs. Thus the development of mGluR3 selective compounds is eagerly awaited and could represent a crucial step towards the potential use of these compounds for use in neurodegenerative conditions such as PD.

## Figures and Tables

**Table 1:** Mean striatal concentrations of dopamine (DA), dihydroxyphenylacetic acid (DOPAC), and homovanillic acid (HVA) as measured by HPLC-ECD in the unlesioned and lesioned striata in each treatment group. Data shown are mean ng/mg wet weight tissue ± s.e.m. *P < .05, **P < .01 unlesioned versus lesioned hemisphere, ^†^ *P* < .05, ^††^ *P* < .01 2*R*,4*R*-APDC versus vehicle-treated animals [10]. Drug concentration in nmol (final injection volume 4 *μ*L).

Treatment group	[DA] Unlesioned ng/mL	[DA] Lesioned ng/mL	[DOPAC] Unlesioned ng/mL	[DOPAC] Lesioned ng/mL	[HVA] Unlesioned ng/mL	[HVA] Lesioned ng/mL
Vehicle	7.26 ± 0.16	1.042 ± 0.23**	1.99 ± 0.13	0.59 ± 0.15**	0.74 ± 0.07	0.33 ± 0.09**
2*R*,4*R*-APDC	7.87 ± 0.18	3.40 ± 0.54^∗∗††^	1.46 ± 0.10	0.81 ± 0.07^∗∗†^	0.65 ± 0.04	0.45 ± 0.06^∗∗†^

**Table 2:** Measurement of striatal dopamine metabolism in the unlesioned and lesioned striata in each treatment group. Data are expressed as mean dopamine turnover ratio ± s.e.m calculated by the expression (DOPAC + HVA/DA) using mean pmol/mg wet weight tissue values for DA, DOPAC, and HVA, respectively. *P < .05, unlesioned versus lesioned hemisphere. ^†^ *P* < .05, 2*R*,4*R*-APDC versus vehicle-treated animal [10]. Drug concentrations in nmol (final injection volume 4 *μ*L).

Treatment group	DA turnover ratio Unlesioned	DA turnover ratio lesioned
Vehicle	0.30 ± 0.04	0.45 ± 0.12*
2*R*,4*R*-APDC	0.26 ± 0.10	0.31 ± 0.04^∗†^

## Nonstandard Abbreviations

2*R*,4*R*-APDC: 2*R*,4*R*-4-aminopyrrolidine-2,4-dicarboxylate
EGLU: (*S*)-*α*-Ethylglutamic acid
DCG-IV: (2*S*,2′*R*,3′*R*)-2-(2′,3′-Dicarboxycyclopropyl)glycine
HPLC-ECD: high performance liquid chromatography- electrochemical detection
LY379268: (1*R*,4*R*,5*S*,6*R*)-4-Amino-2-oxabicyclo[3.1.0]hexane-4,6-dicarboxylic acid
LY354740: (1*S*,2*S*,5*R*,6*S*)-2-aminobicyclo[3.1.0]hexane-2,6-dicarboxylic acid
6-OHDA: 6-hydroxydopamine; mGluR, metabotropic glutamate receptor
MTN: medial terminal nucleus
NMDAR: *N*-methyl-*D*-aspartate receptor
PD: Parkinson's disease
+PHNO: 4-propyl-9-hydroxynaphthoxazine
SNc: substantia nigra pars compacta
SNr: substantia nigra pars reticulata
STN: subthalamic nucleus
VTA: ventral tegmental area.
